# Emergence of a Novel NDM-5-Producing Sequence Type 4523 Klebsiella pneumoniae Strain Causing Bloodstream Infection in China

**DOI:** 10.1128/spectrum.00842-22

**Published:** 2022-08-22

**Authors:** Peiyao Jia, Xinmiao Jia, Ying Zhu, Xiaoyu Liu, Wei Yu, Rui Li, Xue Li, Mei Kang, Yingchun Xu, Qiwen Yang

**Affiliations:** a Department of Clinical Laboratory, State Key Laboratory of Complex Severe and Rare Diseases, Peking Union Medical College Hospital, Chinese Academy of Medical Sciences and Peking Union Medical College, Beijing, People’s Republic of China; b Medical Research Center, State Key Laboratory of Complex Severe and Rare Diseases, Peking Union Medical College Hospital, Chinese Academy of Medical Sciences and Peking Union Medical College, Beijing, People’s Republic of China; c Department of Laboratory Medicine, Hospital of Chengdu University of Traditional Chinese Medicine, Chengdu, People’s Republic of China; d Department of Clinical Laboratory, Beijing Anzhen Hospital, Capital Medical University, Beijing, People’s Republic of China; e Laboratory of Clinical Microbiology, Department of Laboratory Medicine, West China Hospital, Sichuan University, Chengdu, People's Republic of China; Labcorp

**Keywords:** *Klebsiella pneumoniae*, novel sequence type, ST4523, carbapenem-resistant, NDM-5, bloodstream infection

## Abstract

Klebsiella pneumoniae is a significant infectious pathogen that causes bloodstream infections. This study aimed to genetically characterize a novel sequence type 4523 (ST4523) multidrug-resistant (MDR) K. pneumoniae strain recovered from the blood of a 79-year-old Chinese female patient with severe pneumonia and chronic obstructive pulmonary disease who ultimately died of the infection. The susceptibility testing results showed that strain 18SHX180 is nonsusceptible to cephalosporin, carbapenems, combinations of β-lactam and β-lactamase inhibitors, levofloxacin, and colistin and is only susceptible to amikacin. The phylogenetic structure showed that strain 18SHX180 belongs to a novel sequence type, ST4523, and capsule serotype K111. ST4523 is closely related to ST11, the most dominant clone of clinical carbapenem-resistant K. pneumoniae in China. ST4523 has 2 single-base variants in *mdh* and *phoE*. 18SHX180 showed medium virulence in Galleria mellonella and a mouse intraperitoneal infection model. PacBio Sequel and Illumina sequencing were performed to analyze the genetic characterization of 18SHX180, which contains 2 plasmids (pSHX180-NDM5 and pSHX180-1). pSHX180-NDM5 exhibits 86% coverage and 100% identity with 3 *bla*_NDM-5_-carrying plasmids and contains an additional region coding for the *frmRAB* operon, which permits bacteria to sense and detoxify formaldehyde. pSHX180-1 is responsible for the MDR phenotype: it carries 11 categories of genes for antimicrobial resistance [*aadA16*, *aph(3″)-Ib*, *aph(*6*)-Id*, *bla*_SHV-182_, *bla*_TEM-1A_, *qacE*, *aac(6′)-Ib-cr*, *mph*(A), *floR*, *qnrB6*, *arr-3*, *sul*, *sul2*], all of which are associated with transposons and integrons located in three accessory resistance regions. The novel ST4523 K. pneumoniae strain could threaten the control of antimicrobial resistance, and its discovery calls attention to the genetic evolution of bacteria.

**IMPORTANCE** Klebsiella pneumoniae is a significant infectious pathogen causing bloodstream infections. Due to the dissemination of carbapenemase genes, the incidence of carbapenem-resistant K. pneumoniae (CRKP) has increased, with high morbidity and mortality rates in immunocompromised patients. Here, we reported a novel ST4523 *bla*_NDM-5_-bearing CRKP strain initially recovered from a 79-year-old female who died of both a lower respiratory tract infection and bloodstream infection. We also describe the genetic and phenotypic characteristics of this strain. This study provides important insights into the genetic evolution of ST11 K. pneumoniae.

## INTRODUCTION

Klebsiella pneumoniae is a significant opportunistic and nosocomial pathogen that causes meningitis, respiratory tract infections, urinary tract infections, and bloodstream infections (1). After acquiring drug resistance determinants, especially extended-spectrum β-lactamases (ESBLs) and carbapenemase, through plasmids and transposons, some strains of K. pneumoniae have become resistant to most clinical antimicrobial agents, such as cephalosporin, carbapenems, aminoglycoside, and quinolone. Drug-resistant determinants limit antibiotic therapeutic choices and increase morbidity and mortality rates in immunocompromised patients (2–4). The World Health Organization (WHO) and Centers for Disease Control and Prevention (CDC) consider carbapenem-resistant K. pneumoniae (CRKP) to be one of the most urgent public health threats (5, 6).

The most common resistance mechanism in CRKP is the production of carbapenemases, of which K. pneumoniae carbapenemase (KPC) is the most prevalent, followed by the metalloenzyme NDM (7). Recently, the increasing prevalence of NDM, as reported in several studies, has further narrowed clinical antibiotic therapies, since β-lactams and β-lactamase inhibitor combinations show no activity against metalloenzymes (7). Carbapenemase genes are frequently surrounded by small transposable elements that move between plasmids, increasing the incidence of multidrug-resistant (MDR) strains.

Among the CRKP and even CR-hypervirulent K. pneumoniae (CR-hvKP) strains that cause bloodstream infections in China, sequence type 11 (ST11) is the dominant clone, with a high clinical detection rate. ST11 has always harbored genes that cause resistance to several categories of antimicrobial agents, and these genes then contribute to a multidrug-resistant phenotype (8), which threatens patient clinical outcomes.

In this study, we describe the genetic and phenotypic characteristics of a clinical multidrug-resistant *bla*_NDM-5_-bearing K. pneumoniae isolate belonging to the novel ST4523, which is closely related to ST2856 and ST11, the predominant CRKP STs in China. Notably, the patient infected with this strain died of both lower respiratory tract and bloodstream infections.

## RESULTS

### Clinical information.

The clinical isolate 18SHX180 was a K. pneumoniae strain isolated from the blood culture of a 79-year-old female with severe pneumonia, chronic obstructive pulmonary disease, and a bloodstream infection. The patient was admitted to the hospital on 28 February 2018 due to a lower respiratory tract infection caused by K. pneumoniae. During hospitalization, the patient developed a bloodstream infection and a high fever; her body temperature reached over 39°C. When the bloodstream infection occurred, she was admitted to the intensive care unit immediately and treated with sulperazone (cefoperazone-sulbactam, 3 g, every 8 h [q8h], intravenous drip), amikacin (0.5 g, once a day [q.d.], intravenous drip), polymyxin B (500,000 U, q12h), and caspofungin (50 mg, q.d.) from 15 to 31 March. Despite active treatment, the patient passed away on 31 March, and 18SHX180 was isolated from blood culture on that day. Beside the strain from the blood sample, the K. pneumoniae strain was also isolated from the lower respiratory tract.

### MIC determinations.

The K. pneumoniae isolate 18SHX180 had a MDR phenotype. It exhibited nonsusceptibility to multiple antimicrobial agents, including cefepime, ceftazidime, ceftazidime-avibactam, ceftolozane-tazobactam, aztreonam, piperacillin-tazobactam, meropenem, imipenem, imipenem-relebactam, ciprofloxacin, levofloxacin, and colistin, but remained susceptible to amikacin (Table 1).

**TABLE 1:** MICs for 18SHX180 to 16 antimicrobial agents

Antimicrobial agent	MIC (mg/liter)	Clinical breakpoint^*a*^	Interpretation^*a*^
Colistin	≤1	I ≤ 2 R ≥ 4	I
Piperacillin-tazobactam	>64/4	S ≤ 8/4 R ≥ 32/4	R
Amikacin	≤4	S ≤ 16 R ≥ 64	S
Aztreonam	>16	S ≤ 4 R ≥ 16	R
Ceftazidime	>16	S ≤ 4 R ≥ 16	R
Ceftazidime-avibactam	>8/4	S ≤ 8/4 R ≥ 16/4	R
Meropenem	>8	S ≤ 1 R ≥ 4	R
Imipenem	>8	S ≤ 1 R ≥ 4	R
Ertapenem	>4	S ≤ 0.5 R ≥ 2	R
Ciprofloxacin	>2	S ≤ 0.25 R ≥ 1	R
Levofloxacin	>4	S ≤ 0.5 R ≥ 2	R
Cefepime	>16	S ≤ 2 R ≥ 16	R
Imipenem-relebactam	>8	S ≤ 1/4 R ≥ 4/4	R
Ceftolozone-tazobactam	>8	S ≤ 2/4 R ≥ 8/4	R
Cefoxitin	>16	S ≤ 8 R ≥ 32	R
Ceftriaxone	>8	S ≤ 1 R ≥ 4	R

a S, susceptible; I, intermediate; R, resistant.

### Multilocus sequence typing of 18SHX180.

**FIG 1:**
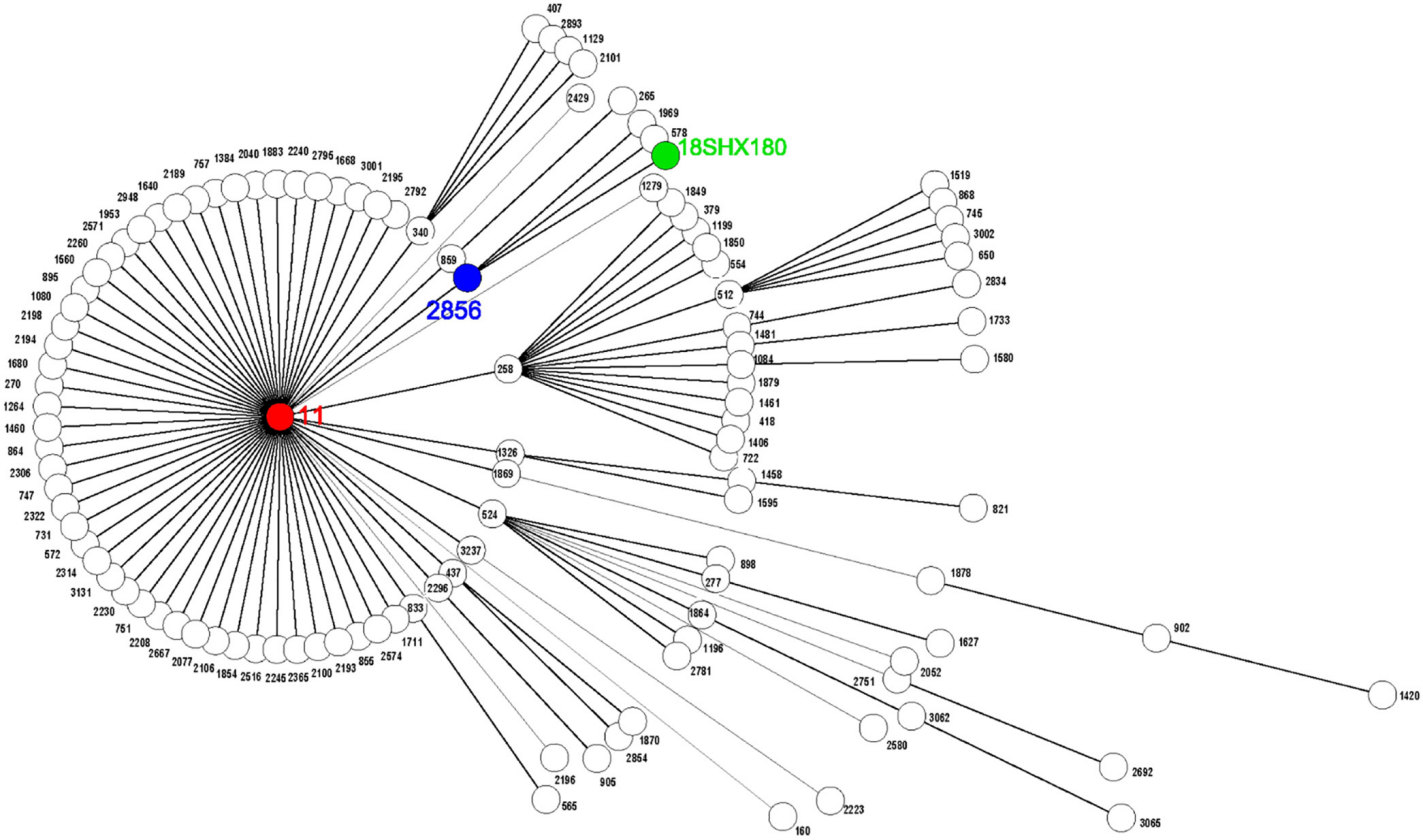
Phylogenic tree of 18SHX180 strains with other ST11 variants. The red node represents ST11, the blue node represents ST2856, and the green node represents 18SHX180. The phylogenetic analysis showed that ST4523 might have evolved from ST2856, a single-locus variant of ST11.

Multilocus sequence typing (MLST) analysis showed that strain 18SHX180 belongs to a novel sequence type, ST4523, and capsule serotype K111. ST4523 was initially discovered in the Department of Clinical Laboratory, Peking Union Medical College Hospital. We compared the loci of housekeeping genes of ST4523 (*gapA*, *infB*, *mdh*, *pgi*, *phoE*, *rpoB*, *tonB*: loci 3, 3, 2, 1, 10, 1, 4) with ST11 (loci 3, 3, 1, 1, 1, 1, 4), and found that *mdh* had mutated from sequence 1 to sequence 2 (due to a C-to-T change at position 429 [C429T]) and *phoE* had mutated from sequence 1 to sequence 10 (C372T). The phylogenetic analysis (Fig. 1) showed that ST4523 might have evolved from ST2856 (loci 3, 3, 2, 1, 1, 1, 4), a single-locus variant of ST11.

### Genomic characteristics and antimicrobial resistance genotype analysis.

**FIG 2:**
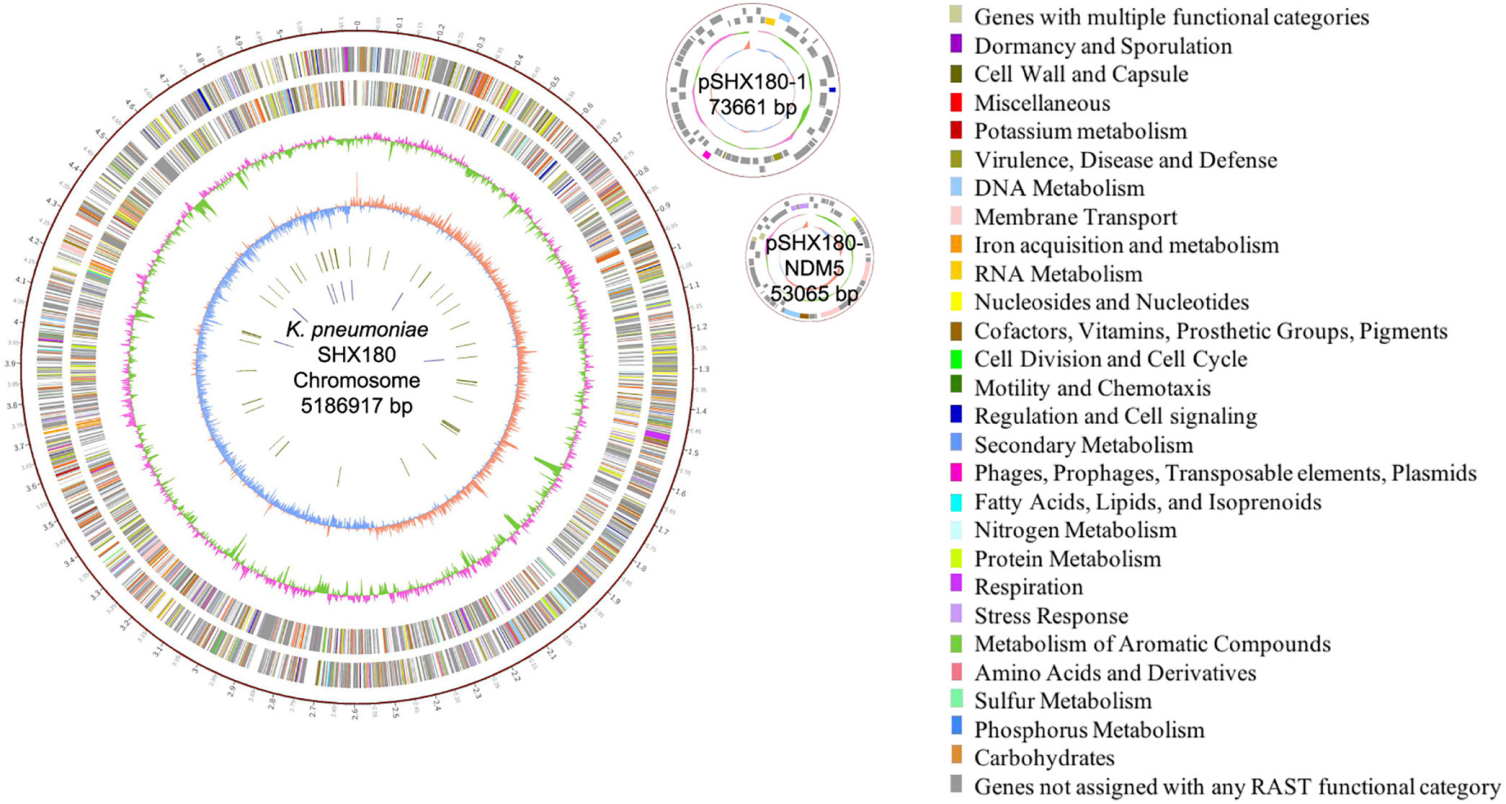
Circular representation of the 18SHX180 genome (chromosome and 2 plasmids). The complete genome of the clinical isolate 18SHX180 is shown, including a 5.2-Mb chromosome, a 74-kb plasmid (pSHX180-1), and a 53-kb plasmid (pSHX180-NDM5). Circles (numbered from outer to inner) report the following: (1) physical map, scaled in megabases from base 1 (start of the putative replication origin); (2) coding sequences transcribed clockwise; (3) coding sequences transcribed counterclockwise; (4) G+C content; (5) GC skew; (6) tRNA genes; (7) rRNA genes. Genes displayed in the coding sequences are color-coded according to functional category, shown on the right.

The complete genome of the clinical isolate 18SHX180 was obtained to investigate the genetic basis of the MDR phenotype by bioinformatic analysis. This strain included a 5.2-Mb chromosome, a 74-kb plasmid (pSHX180-1), and a 53-kb plasmid (pSHX180-NDM5) (Table 2 and Fig. 2). The general chromosome possessed a high GC content (57.69%), 4,955 predicted protein-coding genes with an average gene length of 930 bp, and a high percentage of coding region (88.88%). The 2 plasmids possessed a lower GC content (pSHX180-1, 53.86%; pSHX180-NDM5, 47.12%), a lower ratio of coding regions, and a shorter average gene length (Table 2).

**TABLE 2:** Genomic characteristics and antimicrobial resistance genotype analysis of the sequences of chromosomes and plasmids of 18SHX180

Sequence name^*a*^	Genome size (bp)	GC content	No. of coding genes	Avg gene size (bp)	Coding region bp (% of genome)	tRNA	rRNA	Virulence genes	Drug resistance gene(s)
SHX180-chr	5,186,855	57.69%	4,955	930	4,610,115 (88.88%)	87	25	*iutA, mrkABCDFHIJ, kfuABC, wzi*	*bla*_SHV-182_, *oqxA*, *oqxB*, *fosA*
pSHX180-1	73,661	53.86%	79	758	59,865 (81.27%)	0	0		*mph*(A), *sul1*, *qacE*, *qnrB6*, *aadA16*, *dfrA27*, *ARR-3*, *aac(6′)-Ib-cr*, *floR*, *tet*(A), *bla*_TEM-1A_, *sul2*, *aph(3″)-Ib*, *aph(*6*)-Id*
pSHX180-NDM5	53,065	47.12%	64	698	44,643 (84.13%)	0	0		*bla*_NDM-5_

a Sequences of chromosomes end in chr; sequences of plasmids begin with a p.

Thirteen categories of antimicrobial resistance genes (21 drug resistance genes) were discovered in 18SHX180. There are 3 antimicrobial resistance genes in the chromosome: *oqxAB* (a quinolone and olaquindox efflux pump), the fosfomycin-modifying enzyme-encoding gene *fosA*, and beta-lactam resistance gene *bla*_SHV-182_. A single resistance gene, carbapenemase gene *bla*_NDM-5_, is in the 53-kb plasmid (pSHX180-NDM5). Finally, 11 categories of antimicrobial resistance genes are in the 74-kb plasmid (pSHX180-1), including genes for aminoglycoside resistance [*aadA16*, *aph(3″)-Ib*, *aph*(6*)-Id*], beta-lactam resistance (*bla*_SHV-182_, *bla*_TEM-1A_), disinfectant resistance (*qacE*), fluoroquinolone resistance [*aac(6′)-Ib-cr*], macrolide resistance [*mph*(A)], phenicol resistance (*floR*), quinolone resistance (*qnrB6*), rifampin resistance (*arr-3*), sulfonamide resistance (2 *sul1* and 1 *sul2*), tetracycline resistance [*tet*(A)], and trimethoprim resistance (*dfrA27*).

### Characterization of resistance plasmids.

Genome sequencing showed 2 resistance plasmids, namely, pSHX180-NDM5 and pSHX180-1, in the K. pneumoniae strain 18SHX180, with 64 and 79 predicted open reading frames, respectively.

**FIG 3:**
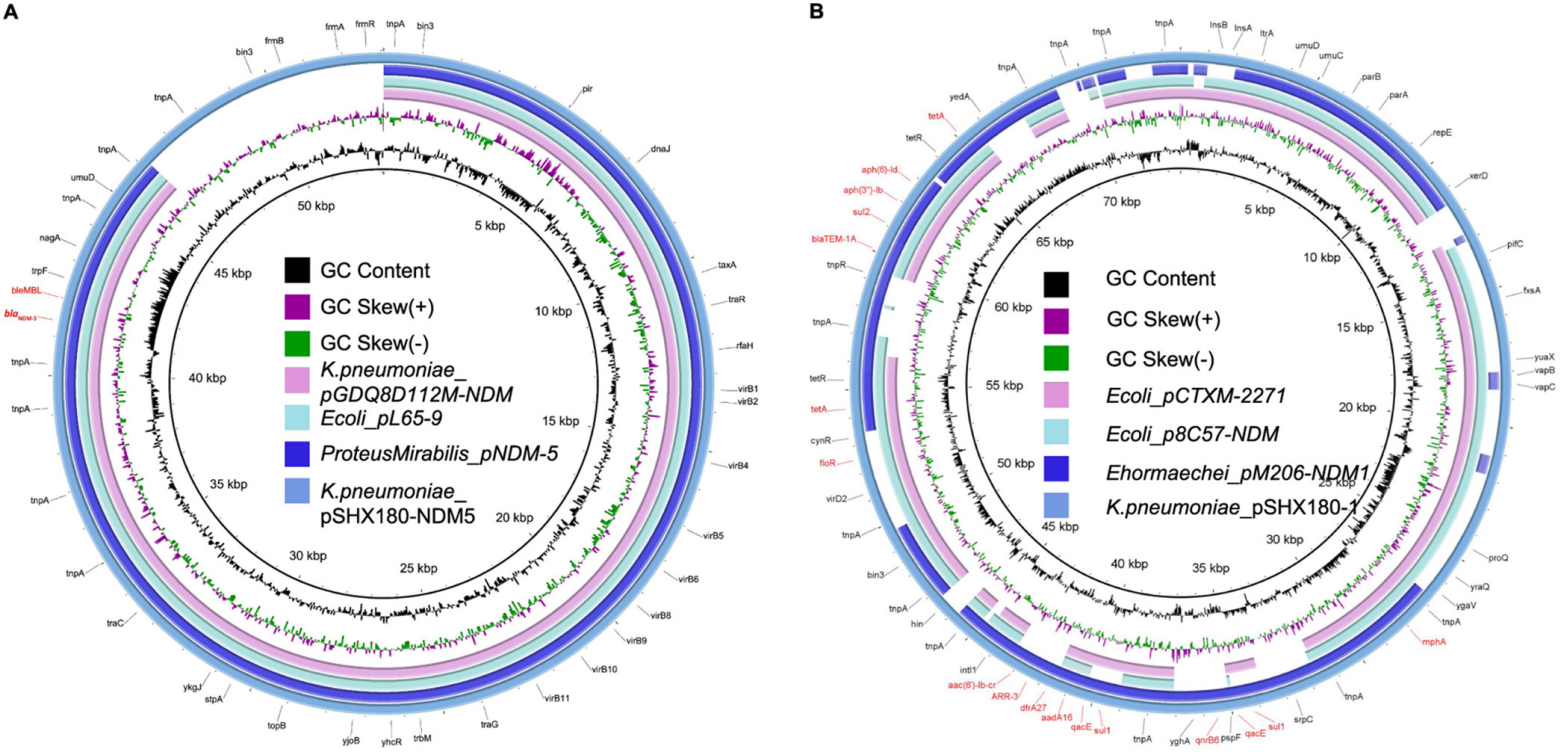
Genetic structure of pSHX180-NDM5 and pSHX180-1. (A) Structural comparison of pSHX180-NDM5 and 3 *bla*_NDM-5_-harboring plasmids (pGDQ8D112M-NDM from K. pneumoniae, pL65-9 from E. coli, and pNDM-5 from P. mirabilis). (B) Structural comparison of pSHX180-1 and 3 multidrug resistance plasmids (p8C57-NDM and pCTXM-2271 from E. coli and pM206-NDM1 from Enterobacter hormaechei). Alignments of plasmids are shown as concentric rings. The outermost rings show the main coding genes of pSHX180-NDM5 and pSHX180-1. Antimicrobial resistance gene names are highlighted in red.

**FIG 4:**
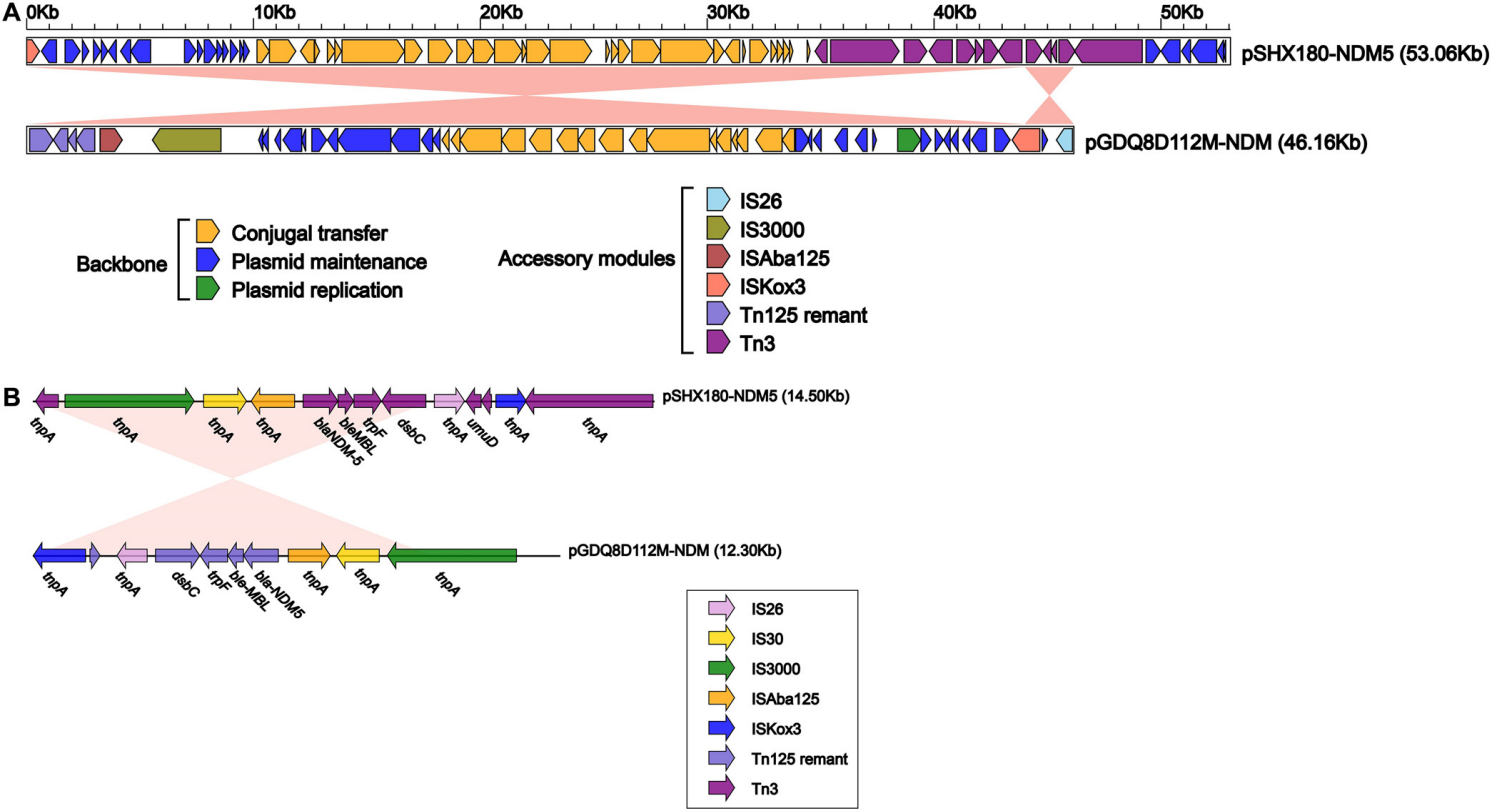
Structure of pSHX180-NDM5 and related transposon. (A) Linear comparison of plasmid pSHX180-NDM5 with the most similar plasmid, pGDQ8D112M-NDM. Arrows denote genes. Genes, mobile elements, and other features are color-coded based on functional classification. Shading denotes the regions with high homology (95% nucleotide identity). (B) Linear genomic comparison of the drug resistance regions in pSHX180-NDM5 and pGDQ8D112M-NDM. The whole region containing the main functional areas of pSHX180-NDM5 is located inside 2 similarly directed TnpA_Tn*3* elements, formatting a TnpA_Tn*3*-based transposon structured as TnpA_Tn3-IS*300*-IS*30*-IS*5*-*bla*_NDM-1_-*ble*_MBL_-*trpF*-*nagA*-IS*26*-*umuD*-IS*Kox3*-TnpA_Tn*3*.

The plasmid pSHX180-NDM5 (53,065 bp) belongs to the IncX3 group and only harbors a resistance gene, *bla*_NDM-5_. Full-plasmid BLAST comparative analysis showed that pSHX180-NDM5 exhibited 86% coverage and 100% identity with 3 *bla*_NDM-5_-carrying plasmids: 1 plasmid of K. pneumoniae (pGDQ8D112M-NDM), 1 of Escherichia coli (pL65-9), and 1 of Proteus mirabilis (pNDM-5) (Fig. 3A). A further linear genomic comparison was conducted between pSHX180-NDM5 and pGDQ8D112M-NDM (*bla*_NDM-5_-carrying plasmid) (Fig. 4), since they share high identity and belong to K. pneumoniae. The backbone region and accessory modules of pSHX180-NDM5 have high similarity to pGDQ8D112M-NDM, with 100% identity and 86% coverage, indicating high-level conservation of the IncX3 backbone. The *bla*_NDM-5_ gene is located in Tn*3* accessory modules, flanked by 2 Tn*3* family transposase elements, and contains IS*3000*, IS*Aba125*, IS*5*, *bla*_NDM-5_, *ble*_MBL_, *trpF* (phosphoribosylanthranilate isomerase), *nagA* (cytochrome c-type biogenesis protein), IS*26*, *umuD* (SOS mutagenesis and repair protein UmuD), and IS*Kox3*. The conjugal transfer elements were also identified in pSHX180-NDM5 through oriTfinder and BLAST, including the transfer origin region (*oriT*), nicking site (*nic*), relaxases, type IV secretion system (T4SS), and type IV coupling proteins (T4CP). The *virB1-11* (excluding *virB3* and *virB7*) and *virD4* genes encode key proteins of a T4SS, and the *virD4* gene also functions as a T4CP. Compared to pGDQ8D112M-NDM, pSHX180-NDM5 contains an additional region encoding the *frmRAB* operon, which permits bacteria to sense and detoxify formaldehyde.

**FIG 5:**
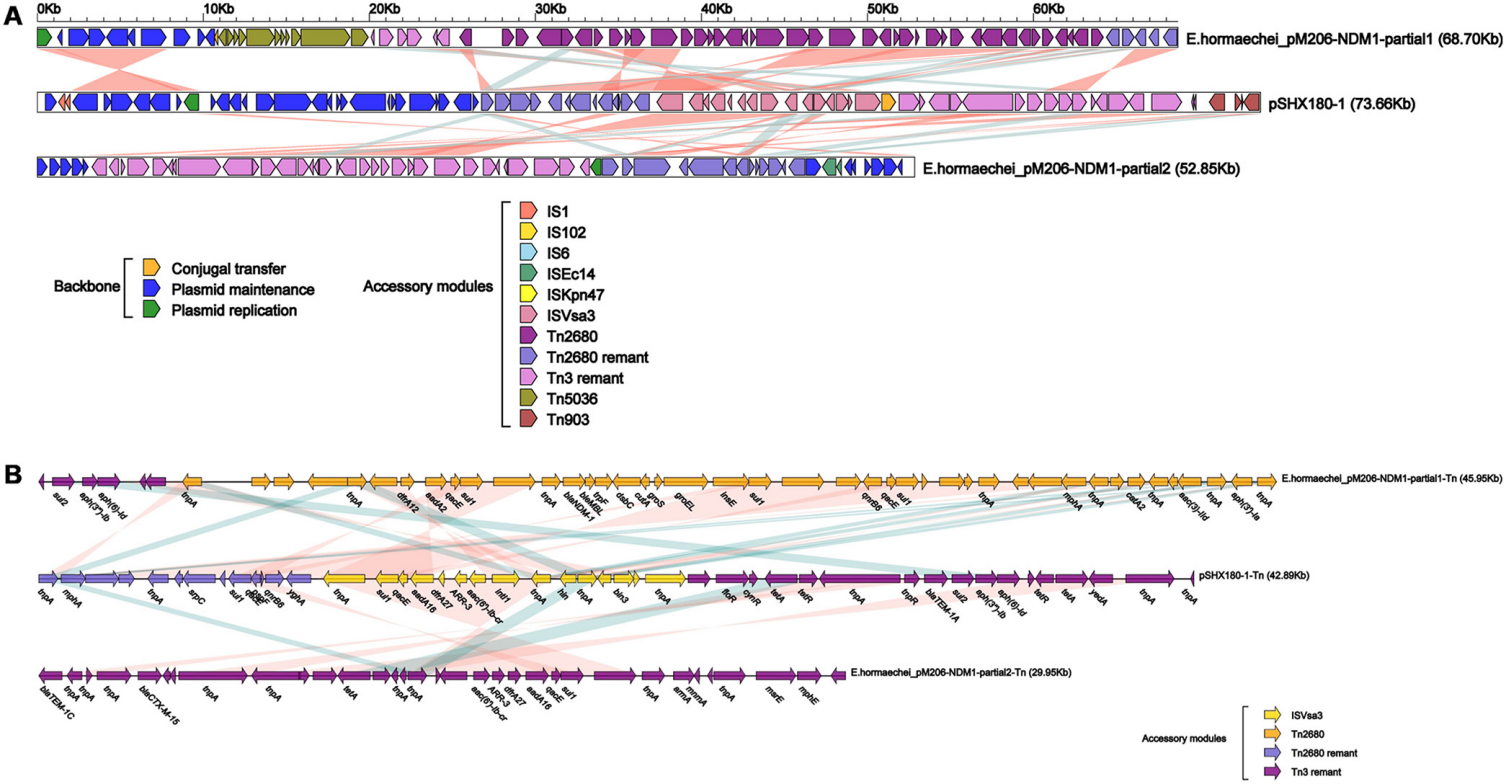
Structure of pSHX180-1 and related transposon. (A) Linear comparison of plasmid pSHX180-1 with plasmid pM206-NDM1, which shares the most similar accessory regions. Arrows denote genes. Genes, mobile elements, and other features are color-coded based on functional classification. Shading denotes the regions with high homology (95% nucleotide identity). (B) Linear genomic comparison of the drug resistance regions in pSHX180-1 and pM206-NDM1. The whole region containing the multidrug resistance areas of pSHX180-NDM5 are located in 3 accessory regions: ΔTn*2680*, IS*Vsa3*, ΔTn*3*.

The other resistance plasmid of K. pneumoniae 18SHX180, pSHX180-1 (73,661 bp in length), belongs to the IncR group and carries multiple drug resistance genes. Sequence analysis showed 99% identity and 70% to 79% coverage with 3 IncR plasmids: 2 plasmids of E. coli (p8C57-NDM and pCTXM-2271) and 1 of Enterobacter hormaechei (pM206-NDM1) (Fig. 3B). Compared to p8C57-NDM and pCTXM-2271, pSHX180-1 contains the same backbone region: a 24.4-kb plasmid maintenance region that contains the replication initiation protein RepE, SOS-regulated proteins UmuD and UmuC, and DNA partitioning proteins ParA and ParB. Since pSHX180-1 and pM206-NDM1 (containing 13 categories of resistance genes and a carbapenemase encoding gene) share high identity in accessory regions, a further linear genomic comparison was conducted between them (Fig. 5). pSHX180-1 contains 5 accessory modules in total. The antimicrobial resistance genes are in the Tn*2680* remnant (ΔTn*2680*), IS*Vsa3*, and the Tn*3* remnant (ΔTn*3*), which are adjacent to the backbone region. No functional genes were found in the other 2 accessory modules; 1 module contains 2 hypothetical proteins flanked by IS*903* elements, and the other contains the IS*1* proteins InsA and InsB.

Regarding the accessory modules, ΔTn*2680* is a partial fragment of the IS*26*-based composite transposon Tn*2680* with an IS*26* element at the 5′ end. The ΔTn*2680* fragment contains a macrolide resistance module, sulfonamide resistance gene *sul2*, quaternary ammonium compound resistance gene *qacE*, and quinolone resistance gene *qnrB6*. The macrolide resistance module encodes macrolide 2′-phosphotransferase, the macrolide resistance major facilitator superfamily transporter Mrx(A), and the macrolide 2′-phosphotransferase I repressor A MphR.

IS*Vsa3* contains another MDR region, which includes *sul1* (sulfonamide resistance), *qacE*, *aadA16* (aminoglycoside resistance), *dfrA27* (trimethoprim resistance), *arr-3* (rifampin resistance), and *aac(6′)-Ib-cr* (fluoroquinolone and aminoglycoside resistance). IS*Vsa3* is flanked by 2 IS*91*-like elements and contains the integron integrase IntI1, *tnp*(A)-phage DNA invertase hin-*tnpA2*, and *bin3*, which has plasmid replication and transfer functions.

The ΔTn*3* region in pSHX180-1 is similar to the corresponding region in the other 3 plasmids (p8C57-NDM, pCTXM-2271, and pM206-NDM1). It also contains 4 categories of antimicrobial resistance genes and only has a transposase element Tn*3* on one side. The Tn*3* remnant is organized sequentially as follows: *floR*, *cynR* (transcriptional regulator), *tet*(A), *tet*(R), *tnp*(A), *tnp*(R), *bla*_TEM-1A_, *sul2*, *aph(3″)-Ib*, *aph(*6*)-Id*, *tet*(R), *tet*(A), *yed*(A) (permease of the drug or metabolite transporter), transpos_Tn*3*, and IS*407*.

### Virulence phenotype and genotype.

**FIG 6:**
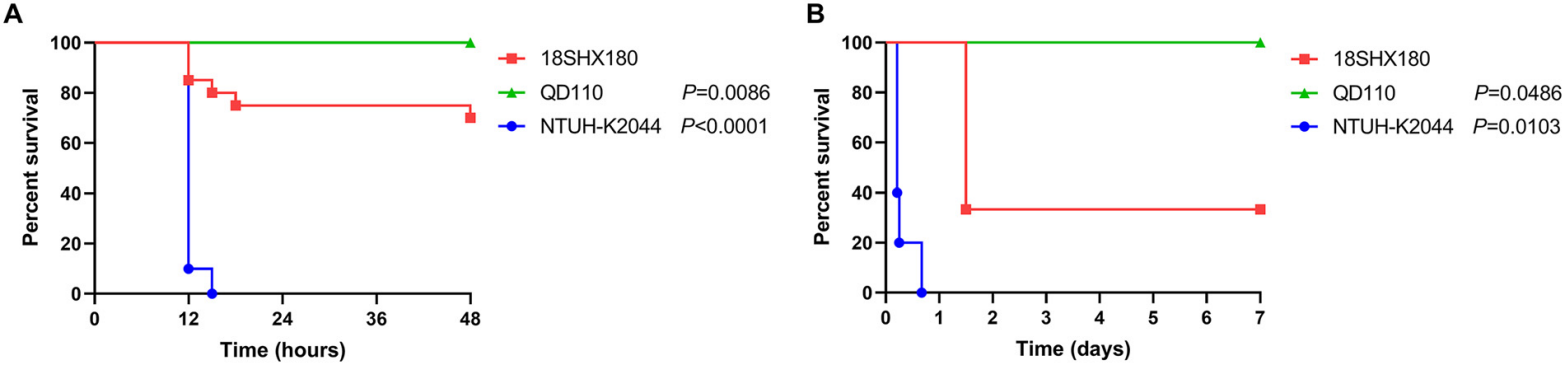
Survival curves that define the virulence phenotype of 18SHX180 observed in the G. mellonella survival assay (A) and mouse intraperitoneal infection model (B). Compared with NTUH-K2044 (a well-known hypervirulent strain used as a hypervirulent control strain) and QD110 (a strain containing no virulence genes in the VFDB, based on the assembled contigs, used as a low-virulence control strain), 18SHX180 showed a medium virulence phenotype in both the G. mellonella survival assay and mouse intraperitoneal infection model.

The analysis of the virulence-associated genes (Table 2) showed that the 18SHX180 strain chromosome carries 4 virulence-associated genes encoding an aerobactin synthesis and transport protein (*iutA*), the fimbriae cluster (*mrkABCDFHIJ*), the ferric uptake system (*kfuABC*), and a capsule assembly protein in the Wzi family (*wzi*). Furthermore, *in vivo*, 18SHX180 showed medium virulence in both the G. mellonella survival assay and mouse intraperitoneal infection model (Fig. 6). Specifically, the survival rates after infection with 18SHX180 were 75% at 18 h in G. mellonella and 30% at 36 h in mice, while the survival rate was 100% after strain QD110 infection and 0% after NTUH-K2044 infection.

## DISCUSSION

The plasmid-mediated spread of multidrug-resistant determinants represents a significant health concern worldwide. In this study, we conducted genetic and phenotypic characterizations of a novel sequence type 4523 carbapenem-resistant K. pneumoniae strain. The novel strain, 18SHX180, with a MDR phenotype, was recovered from a 79-year-old female’s blood sample. ST4523 is a single-locus variant of ST2856 and a 2-loci variant of ST11, which is the predominant sequence type in CRKP and CR-hvKP in China (9). In China, ST2856 has been reported in a clinical CRKP isolate from Zhejiang that caused bloodstream infection and in a clinical CR-hvKP isolate from Chongqing that was only susceptible to tigecycline (10, 11). Similar to the MDR phenotype in ST2856 K. pneumoniae strains, ST4523 K. pneumoniae 18SHX180 from Chengdu was only susceptible to amikacin. Unfortunately, that patient died from the infection due to K. pneumoniae. Epidemiological links of 18SHX180 and ST2856 from Chongqing might be possible, since Chengdu is relatively close to Chongqing. In short, the variants of ST11 are resistant to most clinical antimicrobial agents, especially carbapenem, which is the last line of defense against multidrug-resistant bacteria. It’s concerning that the dissemination of this multidrug-resistant strain might be a threat to infection control and public health. Further study is warranted to track the dissemination of ST4523.

Under antibiotic pressure, bacteria might obtain drug resistance to adapt with phenotypic and genomic changes (12). During hospitalization, the patient in this study was treated with cefoperazone-sulbactam, amikacin, and polymyxin B. However, the isolated K. pneumoniae strain 18SHX180 was resistant to β-lactam agents and aminoglycoside agents, as it harbored two resistance plasmids. pSHX180-1 has 11 categories of resistance genes and mediates multidrug resistance. We conjectured that pSHX180-1 was derived from a fusion: p8C57-NDM (accession number MT407546) supplied the backbone region and pM206-NDM1 supplied the MDR accessory regions. The plasmid pM206-NDM1 contains 13 categories of resistance genes and *bla*_NDM-1_ from an Enterobacter hormaechei strain collected in Japan (accession number AP018830.1) (13). Compared with pM206-NDM1 and p8C57-NDM, pSHX180-1 lacks the *bla*_NDM_ gene, which may have been introduced from an IS*26*-mediated loop-out. The excision of the carbapenemase-encoding gene from a transposon is not rare: partial deletions of this resistance gene were previously reported in K. pneumoniae isolates (14). The partial deletion restored the susceptibility of the strain to carbapenem, and those patients eventually recovered and were discharged (14). However, despite the absence of the *bla*_NDM_ gene in pSHX180-1, 18SHX180 retains a carbapenem resistance phenotype due to the existence of pSHX180-NDM5.

The other plasmid in 18SHX180, pSHX180-NDM5, is identical to pGDQ8D112M-NDM with an addition of the *frmRAB* operon, which could permit bacteria to sense and detoxify formaldehyde and thus escape conventional antibiotic treatments (15). KPC is the dominant mechanism leading to carbapenem resistance in K. pneumoniae, as isolates harboring a *bla*_NDM_ gene generate only a small proportion of CRKP. However, an outbreak of NDM-1-producing ST11 K. pneumoniae strains in a hospital center was reported in Portugal (16), which highlighted the dissemination ability of NDM-producing K. pneumoniae strains. In *Enterobacterales*, the *bla*_NDM-5_ gene has gradually become the main subtype of *bla*_NDM_, and most *bla*_NDM_ genes are carried by the IncX3 plasmid in China, which is in the same plasmid group as pSHX180-NDM5. The IncX3 plasmids are frequently reported to promote the transmission of NDM-5 and have demonstrated high stability (17–20). Type IV secretion system (T4SS) genes, which mediate plasmid conjugation transfer, have also been identified in pSHX180-NDM5, indicating that pSHX180-NDM5 might be self-transmissible and mediate the dissemination of antibiotic resistance. In addition to facilitating plasmid conjugation, T4SS is associated with virulence in Gram-negative organisms (21, 22). In adherent-invasive E. coli, T4SS acts as a principal Escherichia coli colonization factor to form biofilms on the surface of epithelial cells (23).

Genes encoding the fimbriae cluster, the ferric uptake system, and capsule assembly Wzi family protein were found on the chromosome of the 18SHX180 strain. Fimbriae, capsule, and ferric uptake systems are associated with virulence in K. pneumoniae (24, 25). These virulence factors in 18SHX180 could strengthen its pathogenicity, consistent with the medium virulence of 18SHX180 in the G. mellonella survival assay and mouse intraperitoneal infection models. Notoriously, infection is a pathological process caused by the interaction between organism and pathogen under certain conditions. In addition to considering the virulence of these bacteria, we should also consider the patient's physical condition; the patient in this case was elderly with chronic obstructive pulmonary disease. These factors caused the patient’s death within 3 days of fever, which indicated the pathogen invaded the blood, despite active treatment.

In summary, this study characterized the emergence of a novel ST4523 CRKP closely related to ST11. With a multidrug resistance and medium virulence phenotype, this strain may limit clinical therapy strategy and worsen patients’ symptoms and survival. The dissemination of a multidrug-resistant strain may cause great challenges to public health. To prevent the prevalence of multidrug-resistant strains, more research is needed to initiate effective control measures to prevent selection of antibiotic-resistant variants and interrupt the person-to-person or environment-to-person mechanisms of spreading.

## MATERIALS AND METHODS

### Bacterial strains.

The K. pneumoniae isolate 18SHX180 was collected from the blood of a 79-year-old Chinese female patient with severe pneumonia and chronic obstructive pulmonary disease on 31 March 2018. The isolate was identified by matrix-assisted laser desorption ionization–time of flight mass spectrometry.

### Antimicrobial susceptibility testing.

The MICs of colistin, ertapenem, cefoxitin, imipenem, ceftriaxone, piperacillin-tazobactam, ceftazidime, ceftazidime-avibactam, meropenem, ceftolozane-tazobactam, cefepime, amikacin, levofloxacin, aztreonam, and imipenem–MK-7655 were determined by the broth microdilution method. Escherichia coli ATCC 25922 and Pseudomonas aeruginosa ATCC 27853 were used as quality controls. The susceptibilities to antimicrobial agents other than colistin were determined using breakpoints recommended in the CLSI M100 method (26).

### G. mellonella *in vivo* survival assay and mouse intraperitoneal infection model.

To test the virulence of 18SHX180, larval and mouse intraperitoneal infection models were used to compare the survival rates of hvKP reference strain NTUH-K2044 (27) (hypervirulent control strain), the classic K. pneumoniae strain QD110 (9) (low-virulence control strain), and 18SHX180.

For the G. mellonella survival assay (28), standardized 5th- to 6th-instar larvae of 2 to 3 cm in length, good activity, and a creamy color were selected. The larvae were inoculated with 10 μL of bacteria at a concentration of 1 × 10^7^ CFU/mL into the third left proleg. The larvae were observed for survival at 12, 15, 18, 24, 36, and 48 h after bacteria inoculation. Each treatment group had 20 larvae.

A mouse intraperitoneal infection model was generated as previously described with modifications (29, 30). Bacteria were grown in Luria-Bertani broth until logarithmic phase and stored at –80°C after adding an equal volume of 50% glycerinum. Then, the bacteria were washed with phosphate-buffered saline. Three to five CD1 female mice that were 6 or 7 weeks old (Vitalriver) were injected with approximately 5 × 10^7^ to 8 × 10^7^ CFU of NTUH-K2044, QD110, or 18SHX180. The mortality of the mice was observed for up to 7 days.

GraphPad Prism 8 (GraphPad, La Jolla, CA, USA) was used to calculate and present survival rates. The log rank (Mantel-Cox) test was used to compare survival rates between NTUH-K2044, QD110, and 18SHX180. *P* values of <0.05 were considered statistically significant.

### Genomic DNA extraction, sequencing, assembly, correction, and annotation.

The whole-genome sequencing and next-generation sequencing were used to generate 18SHX180’s complete genome. Genome assembly, annotation, and pairwise sequence comparisons were performed following a previously described protocol (31). The genome structure comparisons among plasmids were performed to analyze the sequence homology. Plasmid circular structure maps were produced by BRIG software.

### Sequence analysis scheme.

ResFinder 4.0 was used to identify antimicrobial resistance genes within the whole genomic sequence. Virulence genes were generated from the Virulence Factor Database (VFDB). BLAST was used to search for corresponding genes with 50% coverage and 90% identity by FASTA sequences. The conjugal transfer elements were identified by oriTfinder. STs were determined using SRST2 (32) based on the Illumina reads, whereas serotypes were determined using SerotypeFinder 2.0 (33) based on the assembled contigs. To obtain the phylogenetic features of 18SHX180, we analyzed 18SHX180 and all the MLSTs in K. pneumoniae using Bionumerics.

### Ethical approvals.

The study protocol was reviewed by the Human Research Ethics Committee of the Institutional Review Board (IRB) of the Peking Union Medical College Hospital. This project did not affect the routine diagnosis and treatment of patients; after consultation with the IRB, formal ethical approval was reviewed and waived, and written patient consent was not required (ethics approval number S-K467).

The animal protocols were reviewed and approved by the Animal Ethics Committee and Administration Institutional Animal Care Committee of Tsinghua University. All experiments were carried out in Tsinghua University Animal Biosafety Level 2 under the guidelines of the Ethics of Animal Experimentation Statement.

### Data availability.

The genome data of K. pneumoniae 18SHX180 were deposited in NCBI under BioProject ID PRJNA692086 with accession number CP094512-CP094514.
